# Improving Thoracic Surgery Ward Round Quality and Enhancing Patient Safety in a Referral Centre

**DOI:** 10.7759/cureus.42784

**Published:** 2023-08-01

**Authors:** Mark Boyle, Aina Pons, Abdullah Alshammari, Daniel Kaniu, Asonitis Athanasios, Mohamed Ryan Bashir, Jose Alvarez Gallesio, Hemangi Chavan, Silviu Buderi

**Affiliations:** 1 Department of Surgery and Cancer, Imperial College London, London, GBR; 2 Department of Thoracic Surgery, Royal Brompton Hospital, London, GBR; 3 Department of Cardiac Surgery, Royal Brompton Hospital, London, GBR

**Keywords:** ward round, audit, patient safety, standardisation, note

## Abstract

Introduction

Ward rounds are vital clinical processes that facilitate an opportunity for daily review and management of thoracic surgery inpatients. The aim of this study was to compare thoracic surgery ward round documentation against locally agreed standards and design a template to improve the detail and uniformity of this process to enhance patient care.

Materials and methods

Data for this audit was collected retrospectively and prospectively. Data was collected during three auditing periods and managed on Microsoft Excel. Descriptive statistics were used for its analysis. Chi-square and Fisher’s Exact tests were used to test for differences in reporting rates.

Results and discussion

Initially, a total of 199 ward round notes were reviewed. Imaging results (19%) and discharge planning (23%) were not reported. eCARE (electronic Clinical Assessment for Round Evaluation) was developed to ensure that all aspects of patient evaluation recommended by the guidelines were included. Reporting rates significantly improved after such changes. We analysed the effect of the new ward round note on discharge planning (23.3 vs 41%, p<0.001), complication rates (32.6 vs 21.9%, p=0.03), post-surgical length of stay (LOS) (7.0 vs 5.0, p<0.001).

Conclusion

Over a year, we audited the Thoracic Surgery Department's ward round documentation against locally agreed standards in line with national recommendations. Several important items were not regularly reported. Using closed-ended questions improved reporting rates, and patient care was optimised. Further research should explore the impact of this new documentation method on patient care and postoperative outcomes in our Trust as well as other cardiothoracic centres.

## Introduction

Ward rounds are complex clinical processes that provide an opportunity for daily review and management of surgical inpatients [1]. However, there is considerable variability in the wayward rounds are conducted. The Royal College of Physicians (RCP) and Royal College of Nursing (RCN) jointly set out core recommendations and principles for best practices for conducting medical ward rounds in September 2015 with the view of improving patient care [2].

The National Institute for Health and Care Excellence (NICE) issued in 2017 the Structured Ward Rounds for Emergency and Acute Medical Care in adults [3]. These are all based on clear clinical evidence of benefits in terms of mortality, length of stay (LOS), patient satisfaction, and staff satisfaction. Several authors have investigated the importance of standardisation of routine medical documentation. Some have demonstrated the value of checklists in this process [4]. Others have proven the benefit of early teaching of ward-round documentation on patient safety and care [5]. Another study strengthened that the lack of document uniformity can compromise the quality of medical record-keeping, promote significant variation, and have an impact on handover and continuity of care [6]. The role of ward round documentation is therefore essential, and a structured tool for ward rounds improves communication with patients without prolonging the time of rounding [7].

The documentation of surgical ward rounds is crucial not only for medico-legal reasons but also to ensure continuity of care. Pucher et al. reported that less thorough ward rounds resulted in delayed diagnoses and preventable complications, and negatively affected outcomes [8]. Other authors have highlighted the importance of structured ward rounds to contribute to the education of junior doctors [9]. Good documentation also allows an important forum for collaborative medical reasoning [10]. Additionally, poor-quality ward rounds can lead to a greater number of adverse events, thereby cascading to an increased financial strain on healthcare systems [11].

The aim of this study was to ensure a safer and more uniform ward round in our Thoracic Surgery Department. To this objective, we audited compliance for our service with the guidelines and recommendations issued by the General Medical Council (GMC) and RCP/RCN and developed a system to improve adherence to national ward round strategies.

This article was previously posted to the Research Square preprint server on 6 February 2023.

## Materials and methods

Data for this audit was collected retrospectively and prospectively. The thoracic surgical ward round entries were reviewed from the medical notes by two independent observers. The data was collected during three auditing periods.

From October to November 2021, ward round notes were reviewed without any previous modification. With the help of our IT department, we developed and created eCARE (electronic Clinical Assessment for Round Evaluation) including all mandatory items according to the GMC and RCP/RCN for a high-quality ward round with mostly open-ended questions.

From March to April 2022, we conducted a satisfaction survey amongst members of the thoracic surgical team on the use of the new thoracic surgery ward round note. Taking this feedback into consideration, we designed a new note that would remain user-friendly. Based on previous literature and good outcomes we implemented several checklists to allow more efficient and less time-consuming information gathering during thoracic surgery ward rounds.

From June to July 2022, we reviewed the modified ward round notes and the reporting rates of relevant information and compliance with the guidelines.

Over the three auditing periods, data was managed on Microsoft Excel and descriptive statistics were used for its analysis. Chi-square and Fisher’s Exact tests were used to test for differences in reporting rates. This audit was registered under ID number 004887 in our Cardiothoracic Centre.

## Results

During the first auditing period, a total of 199 patient notes were reviewed and patient details, location, date, time, and ward round lead were reported. All notes were documented chronologically. All notes were signed and the bleep carried by the medical team for further contact was provided. The observations and physical findings were documented in 84% and 80% of the notes. Remarks were added in 92% of the notes. Pain and chest drain observations were reported in 53% and 30% of the notes. The surgical wound was assessed in 16% of all notes. Microbiology results, plan for antibiotics and blood results were reported in 5%, 15% and 24% of all notes respectively. Imaging results (19%) and discharge planning (23%) were often not reported. None of the healthcare professionals documenting the ward rounds included their GMC number (Table 1).

**Table 1:** Reported information during ward rounds compared to guidelines (n=199 patient notes) GMC: General Medical Council

Information	YES (n, %)	NO (n, %)	NA (n, %)
Patient details	199 (100)	0 (0)	0 (0)
Location	199 (100)	0 (0)	0 (0)
Date	199 (100)	0 (0)	0 (0)
Time	199 (100)	0 (0)	0 (0)
Lead	199 (100)	0 (0)	0 (0)
Chronological order	199 (100)	0 (0)	0 (0)
Standardised structure	199 (100)	0 (0)	0 (0)
Observations	168 (84)	31 (16)	0 (0)
Physical findings	160 (80)	37 (19)	2 (1)
Remarks	182 (92)	17 (9)	0 (0)
Pain	105 (53)	85 (43)	9 (5)
Chest drain	59 (30)	31 (16)	109 (55)
Imaging	37 (19)	161 (81)	1 (0)
Blood results	47 (24)	152 (76)	0 (0)
Microbiology results	9 (5)	174 (87)	16 (8)
Surgical Wound	32 (16)	152 (76)	15 (8)
Discharge plan	45 (23)	148 (74)	8 (3)
Antibiotics	30 (15)	156 (78)	13 (7)
Signature	198 (100)	1 (0)	0 (0)
Bleep number	199 (100)	0 (0)	0 (0)
GMC number	0 (0)	189 (95)	10 (5)

After modification in compliance with the guidelines, eCARE was used by the thoracic surgery department for three months. In a satisfaction survey healthcare professionals reported that although the new note did include all valuable information for optimal patient care and was overall useful for the team, the platform was not user-friendly with too many sections and too time-consuming.

After modification of the note, 200 surgical notes in the closed-ended format were reported (Table 2) using eCARE. Reporting rates were significantly improved in documentation of pain and chest drain observations, compared to the previous format (73% vs. 53%, and 58% vs. 30%, respectively, p<0.001). The reporting rates of the results of different investigations such as imaging, blood, and microbiology were significantly higher by using a closed-ended format compared to the open-ended format. Similarly, discharge and antibiotic plans were significantly reported more often using the new format (41% vs. 23%, and 30% vs. 15%, respectively, <0.001). The reporting rates on physical findings and observations were unexpectedly lower compared to the previous format.

**Table 2:** Reporting rates in open-ended versus closed-ended formats (n=200 patient notes) * p-value for two-sided Pearson chi-square. **p-value for two-sided Fisher's Exact test. GMC: General Medical Council

Information	Open-ended format			Closed-ended format			P*
	YES (n, %)	NO (n, %)	NA (n, %)	YES (n, %)	NO (n, %)	NA (n, %)	
Patient details	199 (100)	0 (0)	0 (0)	200 (100)	0 (0)	0 (0)	1
Location	199 (100)	0 (0)	0 (0)	1 (0.5)	199 (99.5)	0 (0)	<0.0001
Date	199 (100)	0 (0)	0 (0)	200 (100)	0 (0)	0 (0)	1
Time	199 (100)	0 (0)	0 (0)	200 (100)	0 (0)	0 (0)	1
Lead	199 (100)	0 (0)	0 (0)	193 (96.5)	7 (3.5)	0 (0)	0.15**
Chronological order	199 (100)	0 (0)	0 (0)	198 (99%)	2 (1%)	0 (0)	0.499**
Standardised structure	199 (100)	0 (0)	0 (0)	177 (88.5)	23 (11.5)	0 (0)	<0.0001
Observations	168 (84)	31 (16)	0 (0)	137 (68.5)	63 (31.5)	0 (0)	<0.0001
Physical findings	160 (80)	37 (19)	2 (1)	124 (62)	76 (38)	0 (0)	<0.0001
Remarks	182 (92)	17 (9)	0 (0)	182 (91)	18 (9)	0 (0)	0.872
Pain	105 (53)	85 (43)	9 (5)	146 (73)	52 (26)	2 (1)	<0.0001
Chest drain	59 (30)	31 (16)	109 (55)	116 (58)	20 (10)	64 (32)	<0.0001
Imaging	37 (19)	161 (81)	1 (0)	131 (65.5)	65 (32.5)	4 (2)	<0.0001
Blood results	47 (24)	152 (76)	0 (0)	120 (60)	79 (39.5)	1 (0.5)	<0.0001
Microbiology results	9 (5)	174 (87)	16 (8)	61 (30.5)	134 (67)	5 (2.5)	<0.0001
Surgical Wound	32 (16)	152 (76)	15 (8)	33 (16.5)	154 (77)	13 (6.5)	0.919
Discharge plan	45 (23)	148 (74)	8 (3)	82 (41)	118 (59)	0 (0)	<0.0001
Antibiotics	30 (15)	156 (78)	13 (7)	59 (29.5)	130 (65)	11 (5.5)	0.003
Signature	198 (100)	1 (0)	0 (0)	200 (0)	0 (0)	0 (0)	0.499**
Bleep number	199 (100)	0 (0)	0 (0)	125 (62.5)	75 (37.5)	0 (0)	<0.0001
GMC number	0 (0)	189 (95)	10 (5)	0 (0)	200 (100)	0 (0)	0.001**

After observing these changes, we finally modified eCARE to ensure that the sections of information that had not been systematically reported were made mandatory. As a result, the guideline recommendations were maintained whilst prioritising patient safety.

To assess the impact of the undertaken measures on patient care we analysed the impact of eCARE on discharge planning, complication rates, post-surgical LOS and mortality (Table 3). When comparing patient outcomes before and after the implementation of the new ward round we concluded that notes discharge planning (23.3% vs. 41%, p<0.001), complication rates (32.6% vs. 21.9%, p=0.03) and LOS (7.0% vs. 5.0, p<0.001) were significantly better after the implementation of the note. Conversely, the mortality difference in the two cohorts was not found to be statistically significant (2% vs. 4.5%, p=16).

**Table 3:** Impact of new ward round note on patient care LOS: length of stay, IQR: inter-quartile range

	Period 1	Period 2	P-value
Discharge planning	45 (23.3%)	82 (41%)	<0.001
Complications	57 (32.6%)	37 (21.9%)	0.03
Post-surgical LOS median (IQR 25-75%)	7.0 (4.0-19.0)	5.0 (3.0-11.0)	<0.001
Alive	195 (98%)	191 (95.5%)	0.16

## Discussion

Our study to design eCARE for the Thoracic Surgery Department has been undertaken in three cycles. The first cycle evaluated the compliance of our ward rounds with GMC and RCS/NCS Guidelines. Although the ward notes included most of the important information for patient care this was not standardised and important items were missing when compared with standards including key information such as drain output and pain assessment. We used this document to design eCARE, a new ward round note that would include all items that were recommended and that would help provide optimal patient care in an electronic, user-friendly format.

The rationale behind the usage of a template for the ward rounds in place of a free text note was based on previous evidence that suggested that when following instructions on a ward round template compliance improved significantly in all parameters when reviewing patients and that this further enhanced patient safety [12]. The usage of a template for ward round documentation improved reporting rates and ensured all important information was asked of the patient and all relevant investigations were reviewed [13]. In another study, the usage of a ward round template increased the amount of information reported and increased the junior doctor's confidence in ward round documentation [14].

The thoracic surgical team used eCARE for three months and was surveyed on its usage afterwards. Although compliant with guidelines and thoroughly focused on patient care, the team reported that the open-ended question format made the note too tedious and difficult to complete in often busy ward rounds. This was consistent with previous research that stated that at least 10 minutes should be dedicated per patient for a routine ward round to ensure good quality of care [15]. Previous research had shown that structuring the ward round notes had had a very positive impact on busy ward rounds, specifically during the COVID-19 pandemic [16] and we applied this evidence to implement a new type of ward round note including checklists and drop lists. We based this implementation on several papers that have been published reinforcing the value of checklists during ward rounds. Wen et al. investigated the role of these and concluded that the checklist can be used to quickly identify the focus of quality inspection and could reduce the incidence of unexpected adverse events [17]. Specifically, another study concluded that checklist-based prompting improved multiple processes of care, including reported improvement in mortality and LOS in their cohort, reducing errors of omission. This study also revealed that the application of the bedside ward round checklist can reduce the incidence of deep vein thrombosis (DVT) in critical patients [18].

**Figure 1:**
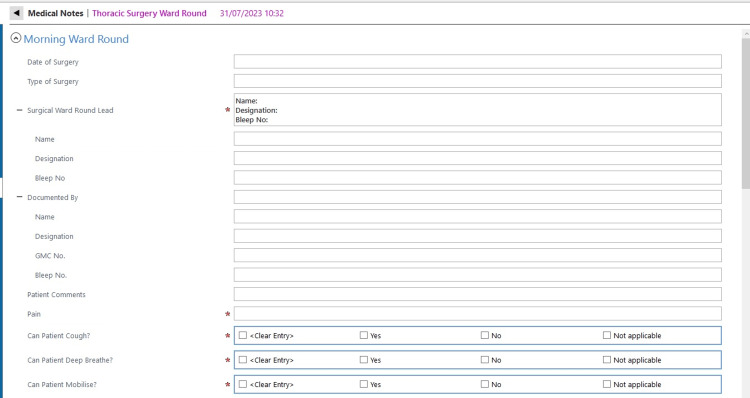
eCARE Thoracic surgery ward round template part A. eCARE: electronic Clinical Assessment for Round Evaluation

**Figure 2:**
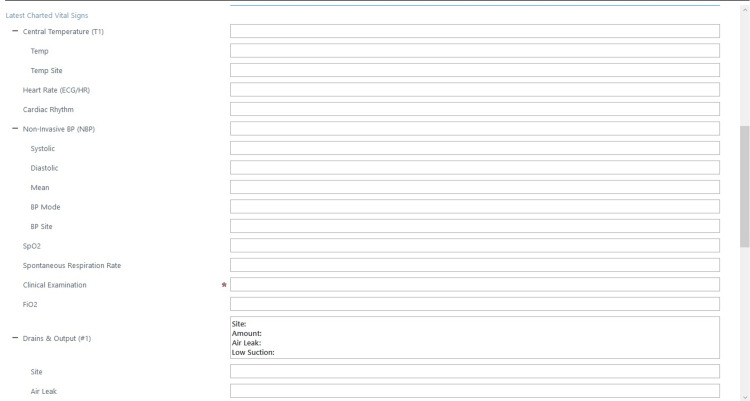
eCARE Thoracic surgery ward round template part B. eCARE: electronic Clinical Assessment for Round Evaluation

**Figure 3:**
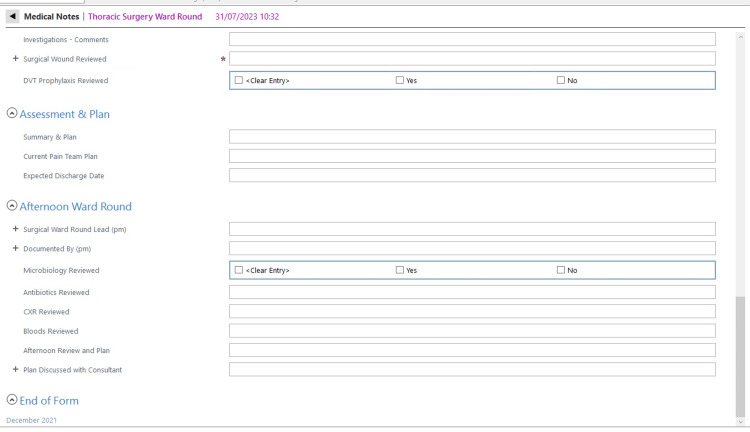
eCARE Thoracic surgery ward round template part C. eCARE: electronic Clinical Assessment for Round Evaluation

We, therefore, implemented a second ward round note adequately formatted and taking this evidence into account. We involved the pain team to design a symptoms checklist to allow efficient and accurate reporting of pain levels in postoperative patients using closed-end questions and a grading scale. Our hypothesis was that using closed-ended questions format would improve reporting rates and clinical documentation. During the second cycle of our audit, we reported the usage of this note with very good results. However, some of the items recommended by the guidelines were still not reported routinely and had therefore been made mandatory on the note. After our last auditing cycle, we have concluded that our note is now compliant with the guidelines. eCARE is a standardised note, using closed-end questions and with some selected mandatory items as been implemented in our Thoracic Surgery Department and could be used by other surgical specialities that seek to document more complete and thorough ward rounds. See Figures 1-3.

The analysis of the impact of more accurate clinical documentation compliant with the guidelines was undertaken by observing if the difference in complication, LOS, mortality, and efficient discharge planning was significant when we compared the results before and after the changes on the ward documentation notes. We compared patient outcomes before and after the auditing period and concluded that when the new documentation note was implemented discharge planning was better, LOS was statistically shorter and complication rates were statistically significantly lower. We hypothesize that a closer follow-up in a structured manner allows monitoring of all relevant aspects of patient care in postoperative patients provided more careful, thorough, and detailed care. This led to superior outcomes whilst shortening LOS also facilitated by more precise and thoughtful discharge planning (planning for discharge being a mandatory item on the new note).

However, when we compared mortality the difference between the two periods was not significant. This could be due to a possible delay in the uptake of this new method on long-term results. Additionally, the time through which we evaluated mortality in the second group was shorter than the first group (as patients were followed up from operation day to present) and this could therefore limit the interpretation of our results.

Based on the benefits of systematic ward rounds and checklists presented by previous research [19,20] and compliantly with national standards we have developed a ward round note that includes all mandatory information for daily patient review. Further research should explore the impact of this new documentation method on patient care and postoperative outcomes.

## Conclusions

Over a year, we audited the thoracic surgery departments' daily ward round documentation against locally agreed standards in line with national guidelines. Several important items were not regularly reported during ward rounds. We created eCARE, a new thoracic ward round note to comply with such standards. Although these changes were satisfactory with improvement in patient care and experience, we hypothesised that using closed-ended questions format would improve reporting rates for surgical notes documentation. Using this format, reporting rates improved, and patient care and safety were optimised. Further research should explore the impact of these new documentation methods and the usage of checklists on patient care and postoperative outcomes in our Trust as well as other cardiothoracic centres.
